# Eye movement characteristics in schizophrenia: A recent update with clinical implications

**DOI:** 10.1002/npr2.12087

**Published:** 2019-11-27

**Authors:** Kentaro Morita, Kenichiro Miura, Kiyoto Kasai, Ryota Hashimoto

**Affiliations:** ^1^ Department of Rehabilitation University of Tokyo Hospital Tokyo Japan; ^2^ Department of Neuropsychiatry Graduate School of Medicine The University of Tokyo Tokyo Japan; ^3^ Department of Pathology of Mental Diseases National Center of Neurology and Psychiatry National Institute of Mental Health Tokyo Japan; ^4^ The University of Tokyo Institutes for Advanced Study (UTIAS) Tokyo Japan; ^5^ Osaka University Osaka Japan

**Keywords:** biomarker, eye movement, eye‐tracking, recovery, schizophrenia

## Abstract

Eye movements are indispensable for the collection of visual information in everyday life. Many findings regarding the neural basis of eye movements have been accumulated from neurophysiological and psychophysical studies. In the field of psychiatry, studies on eye movement characteristics in mental illnesses have been conducted since the early 1900s. Participants with schizophrenia are known to have characteristic eye movements during smooth pursuit, saccade control, and visual search. Recently, studies evaluating eye movement characteristics as biomarkers for schizophrenia have attracted considerable attention. In this article, we review the neurophysiological basis of eye movement control and eye movement characteristics in schizophrenia. Furthermore, we discuss the prospects for eye movements as biomarkers for mental illnesses.

## Abstract 2

In this paper, we review recent research on eye movements in schizophrenia and discuss the prospects for eye movements as biomarkers for mental illnesses.

## Graphical abstract

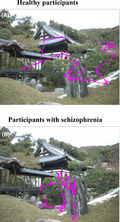

## 1 INTRODUCTION

Visual information is necessary in various situations such as for gathering information at work. In humans, visual acuity progressively decreases from the central to the peripheral visual field. Therefore, to incorporate accurate visual information, it is necessary to bring the image of the visual object to the fovea. Eye movements play an important role in this process.

Eye movements can be measured objectively, and studies have been conducted in various species including humans and nonhuman primates. Studies on nonhuman primates have discovered much about the neurobiological basis of eye movements^1^, ^2^, yet even now, this field is an area of intense research, still leading to new findings.^3^, ^4^ Studies in humans can be conducted in more complex situations in comparison with nonhuman primate studies such as playing table tennis^5^ or viewing pictures with different task instructions.^6^ Eye movement characteristics in subjects with mental illnesses have also been studied, with an objective of uncovering the neurobiological basis of these disorders. In 1908, Diefendorf and Dodge first reported smooth pursuit eye movement characteristics in participants with “dementia praecox” (the current schizophrenia) and “bipolar disorder.”^7^ Various characteristics in eye movements have been revealed since then, and the usefulness of eye movements as neurophysiological biomarkers for schizophrenia has also been suggested.^8^

Currently, noninvasive eye‐tracking systems using video cameras are available. Recent advances in the performance of eye‐tracking cameras allow us to measure eye movements with high temporal and spatial resolution. Thus, researches on the eye movements of subjects with mental illnesses including schizophrenia have been actively conducted. In the following section, we will review the neural basis of eye movement control and the eye movement characteristics of schizophrenia. We will then discuss the prospects for eye movements as biomarkers for mental illnesses.

## 2 EYE MOVEMENT CHARACTERISTICS IN SCHIZOPHRENIA

Modern research on eye movements in the field of psychiatry started with the rediscovery of smooth pursuit eye movement characteristics in schizophrenia.^9^ As research progressed, findings in other characteristics, such as saccade control and exploratory eye movements (voluntary control of a sequence of saccades), have also been discovered.^10^, ^11^ Thus, eye movement characteristics in schizophrenia range from simpler subconscious aspects of eye movement, such as smooth pursuit, to more complex, and cognitive aspects, such as visual search.

Smooth pursuit eye movements occur when viewing a moving object, which keeps the image of the object stabilized on the fovea. Smooth pursuit eye movements function to eliminate visual motion, to avoid retinal blur of a moving target, and to achieve a good view of the object of interest. The cerebral cortex plays an important role in the control of smooth pursuit eye movements.^12^, ^13^, ^14^ Studies in monkeys have revealed that smooth pursuit eye movements are obstructed when the middle temporal area (MT) and the medial superior temporal area (MST) in the superior temporal sulcus are lesioned.^15^, ^16^ These areas involve many neurons that respond to visual motion.^17^, ^18^ The frontal eye field (FEF) also plays an important role in the generation of smooth pursuit. Electrical stimulation of this area triggered ipsiversive smooth pursuit eye movements.^19^ Lesions of this area resulted in reduced and impaired smooth pursuit eye movements.^1^, ^20^, ^21^ In addition, findings suggest that the FEF controls gain (ie, ratio of eye velocity over target velocity) and predicts movement of an object.^22^, ^23^, ^24^ Signals from these cerebral cortices are transmitted to the brainstem and the cerebellum where smooth eye movements are generated.

**Figure 1:**
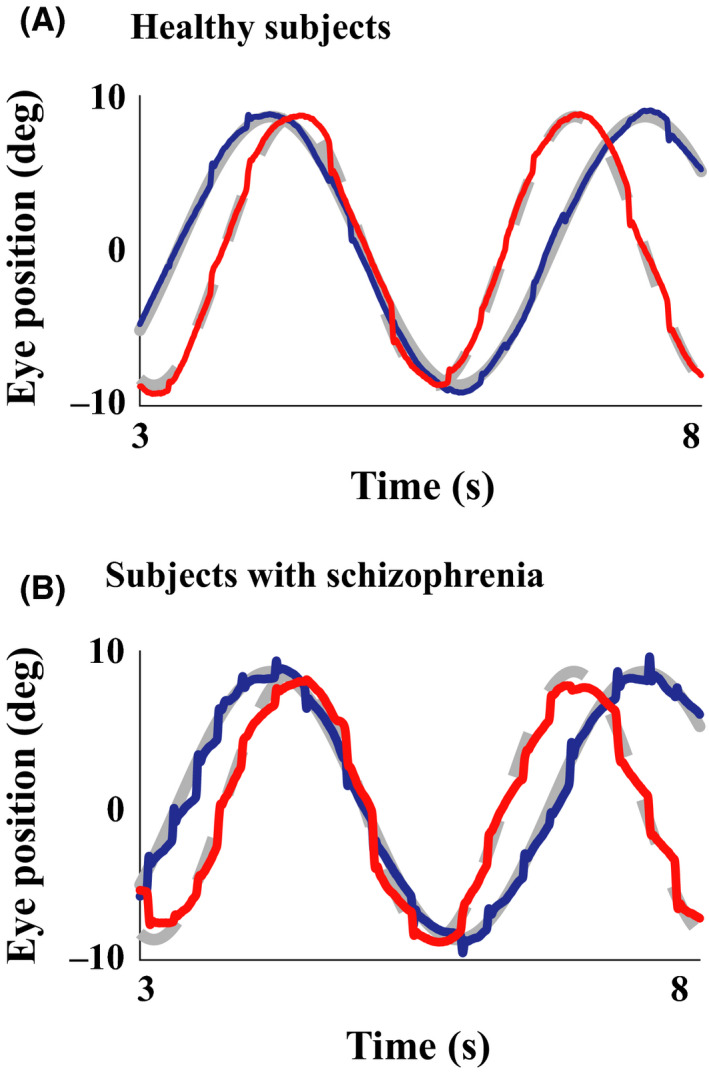
Example eye movement recordings during a smooth pursuit eye movement task. The target positions are indicated by gray lines from the onset of its motion (horizontal direction: gray solid line and vertical direction: gray dashed line), and eye position data are superimposed on them (horizontal: blue lines and vertical: red lines) for a healthy control participant (A) and a participant with schizophrenia (B). Only data from a 5‐s interval are indicated to show the eye movements in detail

Smooth pursuit eye movements are impaired in participants with schizophrenia.^9^, ^25^ When conducting smooth pursuit eye movement tasks, participants are required to track a moving target using their eyes. In example plots of the eye positions during smooth pursuit, healthy participants smoothly follow the visual target (Figure 1A). However, in the participants with schizophrenia, the position of the eye often lags behind the target (especially in the horizontal direction) because the speed of eye movements tends not to keep up with the speed of the moving visual target^26^, ^27^, and catch‐up saccades are seen immediately afterward (Figure 1B). Studies have also shown the possibility that genetic factors are associated with smooth pursuit eye movement characteristics in schizophrenia.^28^, ^29^

Saccades are fast eye movements that bring the image of an object of interest to the fovea. Saccades can occur as an involuntary reflex to suddenly emerging objects, or as voluntary movements to redirect fixation. The superior colliculus (SC) is a brainstem center of saccade control^12^, ^30^, which receives inputs from the cerebral cortex including the lateral intraparietal area (LIP) and the FEF. In particular, the FEF is closely associated with the executive control of saccades, along with the dorsolateral prefrontal cortex (DLPFC) and supplemental eye fields (SEF).^2^, ^31^, ^32^ There are two signal transmission pathways between the FEF and SC. One is a direct excitatory pathway from the FEF to the SC. Another is an indirect pathway via the basal ganglia, where the substantia nigra releases the suppression of SC activity.

**Figure 2:**
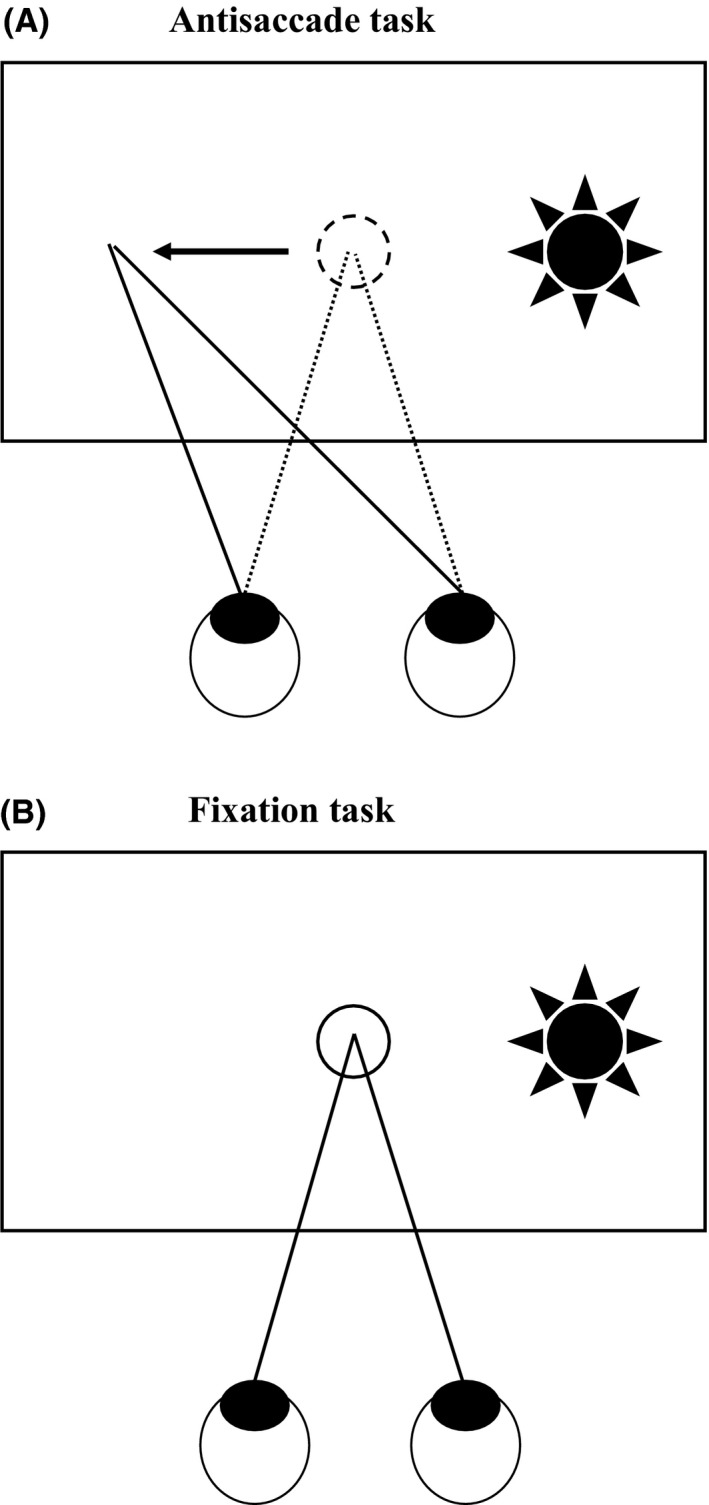
Task designs of saccade control tasks. For the antisaccade task, participants start with fixation on the fixation point in the middle of the screen. Then, when a distractor cue appears, they are instructed to look in the opposite direction of the distractor cue (A). For the fixation stability task, participants are instructed to maintain fixation on the fixation point before and after the distractor cue appears (B)

A task commonly used in the study of eye movements in schizophrenia is the antisaccade task. In this task, when a “distractor” cue emerges, participants are instructed to look in the opposite direction of this cue (Figure 2A). Generally, a visual stimulus that suddenly appears in the scene attracts the observer's attention, and the observer tends to make a saccade toward this distractor cue. Therefore, to achieve this task, the observer needs to inhibit the reflex saccade to the visual cue and make a voluntary saccade to opposite side.

In humans, errors in antisaccade tasks are frequently seen in participants with frontal lobe disorders (especially those with lesions in the DLPFC).^33^, ^34^, ^35^ Fukushima and colleagues first demonstrated that performance of antisaccade tasks (accuracy and latency) was lower in participants with schizophrenia than in healthy participants.^10^ Neuroimaging studies using functional MRI in participants with schizophrenia have also shown that changes in the activation of frontal lesions, such as the DLPFC, are related to increases in errors during the antisaccade task.^36^, ^37^ It has been shown that the effect size of antisaccade characteristics was as large as 1.0 in participants with schizophrenia when compared with healthy participants.^38^ Performance of antisaccade tasks was also significantly heritable within family members related to participants with schizophrenia, and candidate genes have been identified.^39^, ^40^ The ability of response inhibition, which is required for correct antisaccade performance, was also measured by a fixation stability task used by Benson et al.^41^ In this task, participants are instructed to maintain fixation on the fixation point (Figure 2B) while ignoring a distractor cue which appears to the left or right of the fixation point. To succeed in this task, participants must properly suppress reflexive saccades toward the distractor cue. The participants with schizophrenia show more troubles in suppressing reflexive saccades toward the distractor cue, which resulted in shorter duration of fixations than in healthy participants.

**Figure 3:**
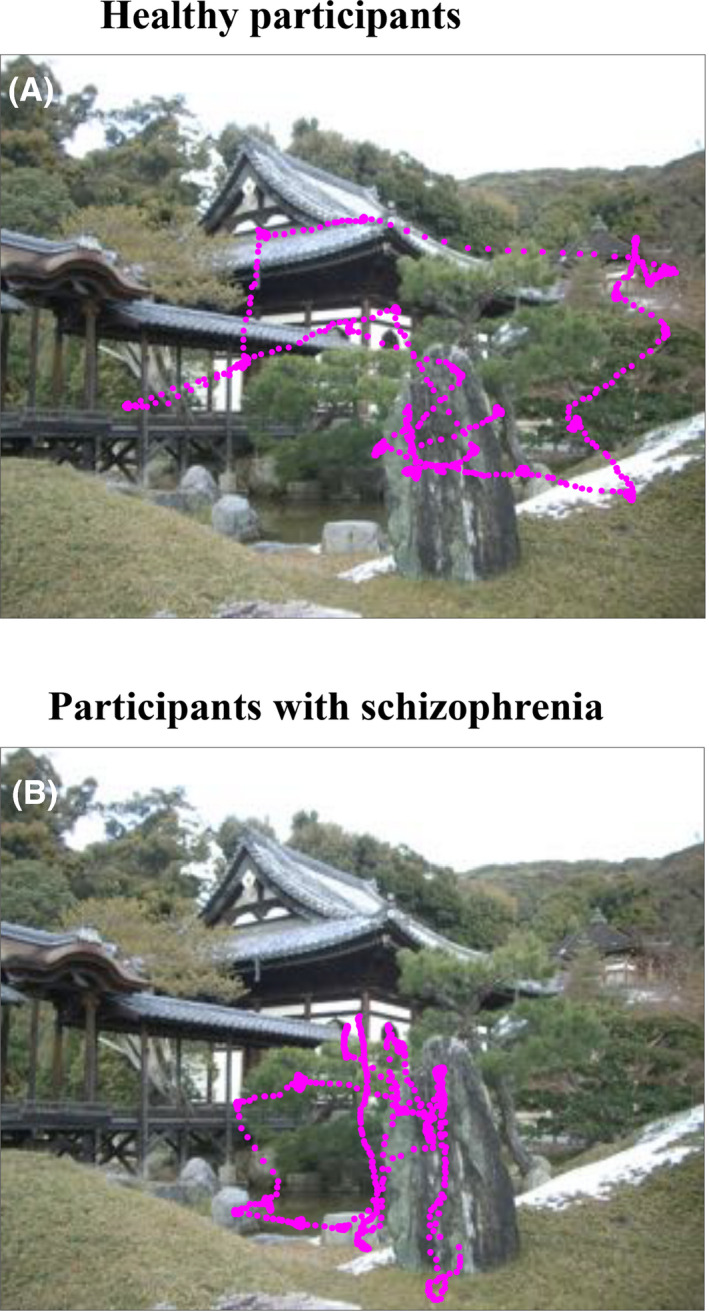
Example eye movement recordings of the free‐viewing task. An example of a picture is shown with eye position data (magenta points) superimposed for both a healthy participant (A) and a participant with schizophrenia (B)

Recent studies have also focused on the exploratory eye movements in schizophrenia. The characteristics of exploratory eye movements are closely related to cognitive processes of individuals^42^, ^43^, and participants with schizophrenia are known to have visuo‐cognitive impairments.^44^ A noteworthy example of research on exploratory eye movements in schizophrenia was conducted by Kojima and Matsushima et al.^11^ They measured eye movements during visual scanning of a lateral S‐shaped figure. First, one S‐shaped figure was shown to the participant, and then, a slightly different target figure was shown. Participants were asked to report the differences between the two figures, and after the answers were revealed, they were asked again to look for any other differences. The participants' exploratory eye movements to the latter question were monitored for 5 seconds. The target figure was divided into seven regions, and a “responsive search score” was calculated depending on how many regions were visited following the prior responses. The responsive search score was found to be smaller in schizophrenia, and various successive studies have been conducted in association with this finding.^45^, ^46^, ^47^ For example, it is known that healthy siblings of schizophrenic participants have significantly lower scores than healthy participants, suggesting a link between the genetic aspects of schizophrenia and responsive search scores.^48^ In a simple free‐viewing task where participants freely view image patterns such as photographs, significant differences in eye movements are seen between participants with schizophrenia and healthy participants. In healthy participants, the eye moves such that the participant's gaze evenly covers the image patterns (Figure 3A). On the other hand, in participants with schizophrenia, the participant's gaze tends to be limited to a smaller area of the photograph (Figure 3B), and the scanpath length is shortened compared with that in healthy participants.^49^, ^50^

## 3 EYE MOVEMENT AS A BIOMARKER FOR SCHIZOPHRENIA

Since various eye movement abnormalities have been shown in schizophrenia, several studies have attempted to develop biomarkers for schizophrenia using eye movement characteristics. Matsushima and Kojima studied the utility of the previously mentioned responsive search score as a discriminator of schizophrenia^45^, which was further extended to a WHO (World Health Organization) collaborative research project involving six countries worldwide. They reported that participants with schizophrenia could be identified from nonschizophrenic participants with a sensitivity of 89% and a specificity of 86.7%.^51^ In addition, they developed an original diagnostic instrument for measuring exploratory eye movements, and a large‐scale multi‐institutional study was conducted in Japan using this instrument. More than 500 participants including healthy participants, participants with schizophrenia, participants with mood disorders, and participants with neurotic and stress‐related disorders participated in their study, and the results showed that they were able to discriminate participants with schizophrenia with a sensitivity of 73.3% and a specificity of 79.2%.^52^

Different eye movements have different neurobiological and mechanistic properties, and by combining different eye movement measures, it would become possible to integrate multiple facets of eye movement characteristics seen in schizophrenia. Arolt et al performed a discriminant analysis of healthy participants and participants with schizophrenia using ten eye movement measures obtained using saccade tasks and smooth pursuit eye movement tasks. They were able to distinguish 90.3% of the participants correctly.^53^ Benson and colleagues used 55 eye movement measures obtained using fixation stability tasks, smooth pursuit eye movement tasks, and free‐viewing tasks, and combined these with machine learning techniques. Their results revealed a high discrimination rate of 98.3%.^41^ The authors of this review have created an integrated eye movement score that represents the eye movement characteristics of schizophrenia using seven eye movement tasks involving fixation stability tasks, smooth pursuit eye movement tasks, and the free‐viewing task. We found that only five eye movement measures were sufficient to distinguish between participants with schizophrenia and healthy participants with a discrimination rate of 89.3%^54^, which makes them relevant in constructing the integrated eye movement score. Morita et al conducted a more thorough study using a larger dataset (85 participants with schizophrenia and 252 healthy participants) and demonstrated a similar discrimination rate (82.5%) using only three eye movement features.^55^ These three measures were each representing different eye movements; horizontal position gain from a fast Lissajous smooth pursuit task, duration of fixations from a saccade control task, and scanpath length from a free‐viewing task. With this improvement, the number of eye movement tasks necessary for obtaining the eye movement score was dramatically reduced in comparison with our first study, and it became possible to perform the whole procedure, from the explanation of eye movement tasks to measurement and analysis, in approximately 30 minutes. Such simplifications are a great advantage of eye movements to obtain biomarkers usable on a clinical basis.

## 4 CURRENT PROBLEMS AND THE ESTABLISHMENT OF LARGE‐SCALE MULTICENTER COLLABORATIONS

It is important that disease biomarkers are both objective, reflecting disease‐specific pathologies, and are beneficial to the individuals with the illness and their supporters. The significant differences seen in eye movement characteristics of schizophrenia, together with the possibility of associated genetic factors, are supportive of a schizophrenia‐specific etiology underlying these eye movement characteristics. However, there are also findings that imply cross‐disorder similarities. Antisaccade errors and shorter scanpath length are known to be seen in other mental illnesses, such as mood disorders^56^, ^57^, and a recent study has shown that genetic factors associated with eye movement characteristics overlap across the spectrum of psychotic disorders.^58^ The neural etiology of eye movement characteristics in schizophrenia is also largely unknown. Previous studies, such as studies examining the association between structural differences in the brain and eye movement measures^59^, ^60^ or those conducting simultaneous measurements of functional MRI and eye movements, have been performed.^61^, ^62^, ^63^, ^64^ However, the findings of these studies were limited by the small sample sizes, and the results were sometimes inconsistent across studies. Therefore, it is still necessary to investigate the specificity of eye movement characteristics of schizophrenia, and the relationship between brain pathology and eye movement characteristics.

**Figure 4:**
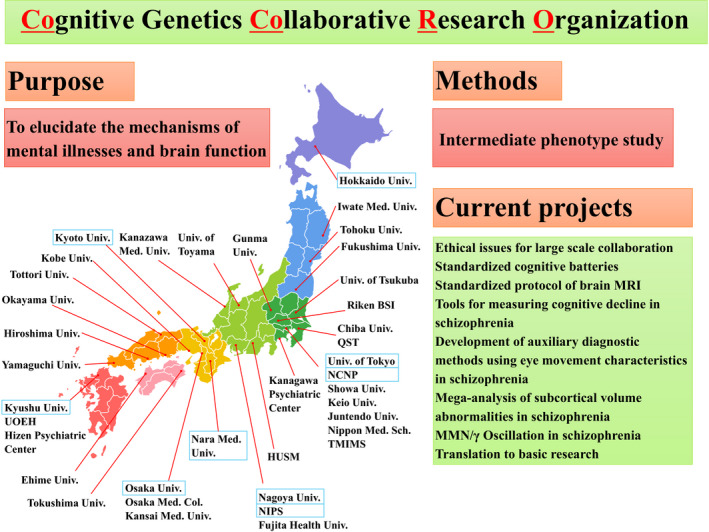
Overview of the COCORO consortium. Thirty‐nine institutions throughout Japan participate in this research consortium. The primary purpose is to elucidate the mechanisms of mental illnesses and brain function by studying intermediate phenotypes. Institutions participating in the multicenter eye movement research project are boxed in blue. Abbreviations: Col.: College; Hosp.: Hospital; Med.: Medical; Sch.: School; Univ.: University; HUSM: Hamamatsu University School of Medicine; NCNP: National Center of Neurology and Psychiatry; NIPS: National Institute for Physiological Sciences; Riken BSI: Riken Brain Science Institute; TMIMS: Tokyo Metropolitan Institute of Medical Science; UOEH: University of Occupational and Environmental Health; and QST: National Institutes for Quantum and Radiological Science and Technology

To achieve such an outcome, large sets of data are necessary. However, there is a limit to how much data a single institution can collect. It is necessary to establish multicenter collaborative research projects where multiple institutions can collect data with standardized protocols and share resources. The pioneer work by Kojima and colleagues described above are good examples, of such an attempt.^51^, ^52^ The Consortium on the Genetics of Schizophrenia (COGS) study explored the link between genes and eye movement characteristics (antisaccade performance) as an intermediate phenotype candidate of schizophrenia.^65^ The Bipolar‐Schizophrenia Network on Intermediate Phenotypes (B‐SNIPS) Consortium was formed to examine intermediate phenotypes involving smooth pursuit eye movement measures and saccade control measures across psychotic disorders including schizophrenia, schizoaffective disorder, and bipolar disorder with psychotic symptoms.^66^ Their study included more than 1000 participants and showed that impairments of smooth pursuit were larger in participants with schizophrenia than participants with the other disorders.^27^ In Japan, the Cognitive Genetics Collaborative Research Organization (COCORO) consortium (Figure 4) has recently been organized, and multicenter eye movement research is currently running within this framework. Multiple centers are cooperating by standardizing data acquisition methods, training inspection methods, and uniformly managing data quality. In addition to eye movements, we have also acquired multimodality data ranging from clinical indicators and cognitive function evaluation scales to neuroimaging data.^67^, ^68^, ^69^, ^70^ We expect further research developments in the near future.

## 5 FUTURE CLINICAL IMPLEMENTATIONS

The collection of visual information is important in everyday life. However, only a few studies have examined the relationship between eye movements and social/cognitive aspects of mental illness.^71^, ^72^, ^73^, ^74^ Not only clinical symptoms but also social/cognitive impairments are known to be a cause of great distress in mental illness and are important factors in therapeutic interventions.^75^, ^76^ We have recently reported eye movement characteristics of schizophrenia and their relationship with cognitive^77^ and social measures.^78^ These studies have shown that in schizophrenia, eye movement measures such as scanpath length, visual cognition such as perceptual organization, and social functioning measured by total work hours per week may have a hierarchical relationship, where eye movement characteristics lead to changes in cognition or social functioning. However, this still needs to be studied in a longitudinal study.

Such findings are important because findings of hierarchical relationships between measures and social functioning have led to the development of new treatment options. For example, associations between cognitive measures and social functioning^79^, ^80^ have led to the development of cognitive treatment programs aiming improve functional recovery.^81^ Mismatch negativity, auditory cognition, and social functioning are also known to have a hierarchical relationship^82^, which have led to studies using mismatch negativity as an index of auditory cognitive training.^83^, ^84^, ^85^ Eye movement measures may also hold such future roles in the treatment in schizophrenia. Some eye movement characteristics, such as exploratory eye movements, are known to change with development^86^ and can be changed with reinforcement learning.^87^ Perhaps by combining findings from recent mathematical models of visual search and exploration^88^, ^89^, ^90^, development of “eye movement training programs” for schizophrenia aimed at improving visual cognition or social functioning may become possible in the future. The development of biomarkers that can be used for clinical and personal recovery would be of great benefit for both the individual with mental illness and their supporters.

## CONFLICT OF INTEREST

The authors declare that they have no conflicts of interest.

## AUTHOR CONTRIBUTIONS

All authors have made substantial contributions to the conception of the work and drafting of the document, have approved of the final version of the draft to be published, and have agreed to be accountable for all aspects of the work.
